# The use of whole-exome sequencing to disentangle complex phenotypes

**DOI:** 10.1038/ejhg.2015.121

**Published:** 2015-06-10

**Authors:** Hywel J Williams, John R Hurst, Louise Ocaka, Chela James, Caroline Pao, Estelle Chanudet, Francesco Lescai, Horia C Stanescu, Robert Kleta, GOSgene, Elisabeth Rosser, Chiara Bacchelli, Philip Beales

**Affiliations:** 1 Centre for Translational Omics – GOSgene, UCL Institute of Child Health, University College London, London, UK; 2 UCL Respiratory Medicine, UCL Medical School, London, UK; 3 Paediatric Respiratory Medicine, Royal London Hospital, Whitechapel, London, UK; 4 Department of Clinical Genetics, Great Ormond St Hospital, London, UK

## Abstract

The success of whole-exome sequencing to identify mutations causing single-gene disorders has been well documented. In contrast whole-exome sequencing has so far had limited success in the identification of variants causing more complex phenotypes that seem unlikely to be due to the disruption of a single gene. We describe a family where two male offspring of healthy first cousin parents present a complex phenotype consisting of peripheral neuropathy and bronchiectasis that has not been described previously in the literature. Due to the fact that both children had the same problems in the context of parental consanguinity we hypothesised illness resulted from either X-linked or autosomal recessive inheritance. Through the use of whole-exome sequencing we were able to simplify this complex phenotype and identified a causative mutation (p.R1070*) in the gene *periaxin* (*PRX*), a gene previously shown to cause peripheral neuropathy (Dejerine–Sottas syndrome) when this mutation is present. For the bronchiectasis phenotype we were unable to identify a causal single mutation or compound heterozygote, reflecting the heterogeneous nature of this phenotype. In conclusion, in this study we show that whole-exome sequencing has the power to disentangle complex phenotypes through the identification of causative genetic mutations for distinct clinical disorders that were previously masked.

## Introduction

**Figure 1:**
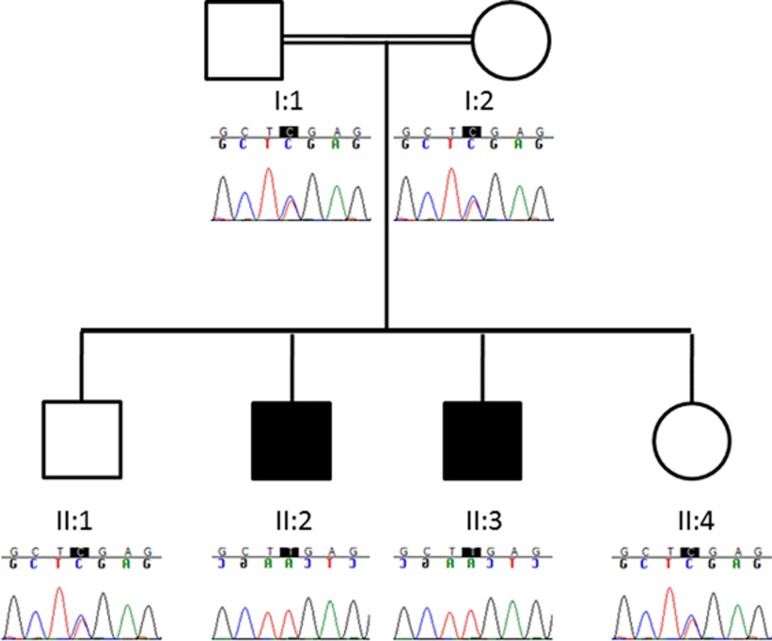
Segregation of PRX variant R1070* in consanguineous Bangladeshi pedigree. Electropherograms generated by Sanger sequencing. Affected siblings (II:2 and II:3) are homozygous T (c.3208C>T; p.R1070*), unaffected siblings (II:1 and II:4) and parents (I:1 and I:2) are heterozygous C/T.

The utility of whole-exome sequencing (WES) for the identification of known and novel disease genes in families segregating rare Mendelian forms of disease is now well established. However, this technique is not as frequently applied to the study of nonsyndromic forms of rare diseases where the phenotypes are more heterogeneous and it is unclear whether they result from the action of a single gene, multiple genes or a complex interaction between genetic and environmental factors. There is therefore a need to extend the scope of WES to the wider patient population so that they can benefit from improved diagnoses and potential therapeutics. To this end we present the data from our WES analysis of a consanguineous pedigree from Bangladesh (Figure 1) diagnosed with a complex phenotype of peripheral neuropathy and bronchiectasis, a phenotype that has not previously been described in the literature.

Peripheral neuropathies are a heterogeneous group of phenotypes that may be isolated or multiple. They may have nongenetic causes such as trauma, toxins or diabetes. Sensory, motor, autonomic nerves may be affected, and there is a complex classification of different neuropathies. However, most cases of early-onset neuropathies affecting many nerves have an underlying genetic cause. More than 500 different genetic conditions causing peripheral neuropathy are listed on the London Medical Database. Similarly, bronchiectasis is a term used to describe a clinicopathological phenotype with multiple routes to causation. These include local causes such as damage due to previous infection, foreign body or developmental abnormality, and systemic conditions which include genetic (such as cystic fibrosis), immunodeficient (principally antibody deficiency) and systemic inflammatory states (such as rheumatoid arthritis). Because of this complexity, even with intensive investigation, in many cases there is no known identifiable cause.

For this family we hypothesised that, due to the presence of consanguinity in the parents and the phenotype being present only in the male offspring the mode of inheritance would be either X-linked or autosomal recessive. In an attempt to identify the causative variant(s) we performed WES in two of the offsprings, one affected and one unaffected (Figure 1; II:2 and II:4, respectively).

## Materials and methods

### Clinical diagnosis

Regarding respiratory manifestations, the older sibling (Figure 1, II.3) had a high-resolution computerised tomography scan diagnostic of bronchiectasis following recurrent chest infections that were slow to clear, and with persistent changes visible on a chest X-ray. Following an admission to hospital with RSV bronchiolitis, the younger sibling (II.2) also had a diagnostic high-resolution computerised tomography performed (requested given the known bronchiectasis in his brother). Both have been extensively investigated in line with current British Thoracic Society guidelines. The clinical diagnosis is therefore one of bronchiectasis likely secondary to aspiration and gastro-oesophageal reflux. Neither has evidence of airflow obstruction (normal FEF25-75, with FEV1 at 90% and 79% for the older and younger sibling, respectively) and both are clinically stable with regular chest physiotherapy, azithromycin antibiotic prophylaxis and acid suppression with omeprazole.

The neuropathy phenotype was first described in sibling II.2 at 20 months of age after he was examined by a clinician due to the observation that he was not walking but showed no sign of being dysmorphic, having hypotonia or joint laxity, concerns persisted as he did not start walking until 34 months of age. He also displayed motor difficulties, such as holding a pen, and was struggling in school. Nerve conduction studies showed a severe demyelinating polyneuropathy with slow conduction velocities and a nerve biopsy demonstrated primary demyelination with onion bulb formation. An MRI scan did not show any definite pathology. Myelination and other metabolic investigations have all been normal whereas genetic testing for chromosome 17q duplication, P0 and gap junction, protein beta-1 were all normal. The younger sibling (II.3) displays a similar phenotype, he did not walk until after 24 months of age, he also had difficulties in school and although he did not have nerve conduction studies, the clinical presentation between the two siblings is undoubtedly the result of a shared condition.

### Linkage analysis

Multipoint parametric linkage analysis was performed on all family members as described previously.^1^

### WES

WES was performed in house using the Agilent SureSelect v1 (38 Mb) kit (II:2) and the Agilent SureSelect v2 (44 Mb) kit (II:4) (Agilent, Santa Clara, CA, USA) followed by sequencing by Illumina GAIIx (Illumina, San Diego, CA, USA; UCL Genomics, London, UK) as 2 × 75 bp runs, one sample per flowcell lane. Data were aligned to genome build hg19 using Novoalign v2 with GATK^2^ used for base quality score recalibration, indel realignment, duplicate removal, and to perform SNP and INDEL discovery and genotyping using standard hard filtering parameters and variant quality score recalibration.^3^

The resultant vcf files from both individuals (case (II.2) versus control (II.4)) were analysed using the Ingenuity Variant Analysis software (Ingenuity, Redwood City, CA, USA) as described in Table 1.

Following the filtering stage the remaining variants were individually assessed to ensure they were not located within a segmental duplication and finally, the bam files were inspected using Integrative Genomics Viewer^4^ software to confirm the quality of the call.

### Sanger sequencing

Standard Sanger sequencing using BigDye v3 chemistry (AppliedBiosystems, Foster City, CC, USA) and Sequencher analysis software (Gene Codes Corporation, Ann Arbour, MI, USA) were used to confirm the presence of each potentially causative mutation and to perform segregation analysis.

### Variant data submission

Verified disease causing variants were submitted to the ClinVar database (http://www.ncbi.nlm.nih.gov/clinvar/).

## Results

Multipoint linkage analysis identified four regions of linkage LOD >2.0 (Supplementary Table 1 and Supplementary Figure 2).

Through WES we generated an average of 2.7 gigabases of sequence and coverage of 70 × across the exons of each individual (Supplementary Table 2). Using Ingenuity Variant Analysis software with the filters described (Table 1) we were able to filter the number of variants from an initial total of 357 699 to a final list of 1 and 13 variants for the peripheral neuropathy and bronchiectasis phenotype analyses, respectively. Removal of variants from within segmental duplications and bad quality calls resulted in a final list of four variants; one unique to the peripheral neuropathy phenotype and three for the bronchiectasis phenotype (Table 1).

Of the remaining variants only two were located within genes functionally related to the phenotype under study; these were *periaxin* (*PRX*) for the peripheral neuropathy and *Interleukin-33* (*IL33*) for the bronchiectasis. The mutation (ClinVar: SCV000212112) in *PRX* was a homozygous nonsense mutation (transcript NM_181882.2; c.3208C>T; p.R1070*) which was not present in the databases of the 1000Genomes,^5^ EVS (http://evs.gs.washington.edu/EVS/) or within an internal database of 451 exomes, however, a single heterozygous carrier was seen in the ExAC database (http://exac.broadinstitute.org; November 2014) giving an estimated allele frequency in 61 486 samples of 8.1 × 10^-6^. The mutation lies within the PRX domain of the protein and has previously been described in the literature (chr19.hg19:g.40901051; rs104894708) to cause Charcot–Marie–Tooth disease type 4 F (CMT4F) and Dejerine–Sottas disease.^6, 7, 8, 9, 10, 11^ Sanger sequencing confirmed the PRX mutation and showed it segregated with disease in the family in a recessive manner (Figure 1).

The variant (chr9.hg19:g.6254555) located within the *IL33* gene consisted of an A nucleotide insertion (transcript NM_033439.3: c.612+2_612+3insA) that was located 2 bp into intron 7 and was predicted to be a potential splice-site mutation. On closer inspection of this region it became clear that this insertion was located adjacent to a homopolymer stretch (poly A) and that there were a number of polymorphisms flanking this repeat within the dbSNP. Homopolymer runs are notoriously difficult to map and can result in misaligned reads, for that reason we chose to Sanger sequence all family members for this region. The Sanger results (Supplementary Figure 1) revealed that the splice-site mutation (rs113609242) was a false positive result which was probably caused by the presence of a variant (rs10975521; transcript NM_033439.3: c612+15 T>A) at position chr9.hg19:g.6254568 located at the opposite end of the homopolymer run. This variant was homozygous non-reference in both the affected individuals and heterozygous in all the unaffected family members but the public databases showed it had a population frequency of >20%, ruling it out as a causative variant. For completeness, we also reviewed all 129 variants from stage IV of the filtering cascade but none were strong candidates for this phenotype.

## Discussion

We present here the results of a study that aimed to identify the genetic cause of a novel phenotype that included peripheral neuropathy and bronchiectasis. We chose to use a combination of linkage analysis and WES to identify the variant due its well documented success in similar circumstances.^12^

Through this approach we were able to identify what we believe to be the causative variant (R1070*), in the gene *PRX*, for the peripheral neuropathy part of the phenotype displayed in this family. Also, as *PRX* is located within a region of positive linkage (LOD=2.1; Supplementary Table 1) on chromosome 19q13.2 we are able to rule out the possibility that this part of the phenotype is due to an X-linked variant.

The gene *PRX* codes for periaxin which encodes two isoforms, L- and S-periaxin, which are structural membrane-associated proteins mainly expressed by myelinating Schwann cells in the peripheral nervous system (PNS). They are required for proper maintenance of the peripheral myelin sheath and Schwann cell compartmentalisation with their integral role reflected in the observation that they account for 16% of PNS myelin protein by weight.^13^ In PRX-null mice the nerve fibres appear grossly normal but there is disruption of the cytoplasmic bands (Cajal bands) and impaired Schwann cell elongation during nerve growth which leads to a reduction in nerve conduction velocity and causes focal hypermyelination and demyelination which are hallmarks of the CMT4F phenotype.^14^

A number of *PRX* variants have been described in the literature,^6, 7, 9^ including the variant we identified (R1070*).^8, 10, 11^ To date the R1070* variant has been found in a number of Japanese CMT4F patients and in one Turkish patient but this is the first report of variant in a patient of Bangladeshi origin.

Our analysis of the *IL33* variant (rs113609242) showed that this was a false positive result. This finding highlights the challenges that still persist in WES analyses and demonstrates the need to verify any potential variant with a second method.

That we were unable to identify a causative variant to describe the presence of the bronchiectasis phenotype is not entirely surprising, the term bronchiectasis refers to a syndrome that has numerous potential causes most of which are not identifiable through WES on a single family.

In summary we demonstrate the utility of WES to disentangle a complex disease phenotype of peripheral neuropathy and bronchiectasis. We have identified the causative variant (R1070*) in the gene *PRX* that is a known cause of Dejerine–Sottas syndrome, which explains the peripheral neuropathy phenotype; however, we have been unable to identify a single causative variant for the bronchiectasis phenotype and suggest that this likely reflects the complex aetiology of this phenotype.

This finding offers hope to those patients who do not exhibit a classic disease phenotype, for whatever reason, that in some cases, through the application of WES, their phenotype can be broken down into its constitutive parts and causative variants can be found that explain specific features. The identification of these specific phenotypic features will allow their targeted treatment, where therapies are available, which will have a positive impact on patient care and more generally it will lead to a better understanding of the illness holistically. We believe there are likely to be a great many patients who are currently undiagnosed who would benefit from the use of WES to disentangle their complex phenotypes to identify known disease genes. The potential to improve the prognosis of such patients makes the use of WES a very cost-effective strategy in the clinic.^15^

## Figures and Tables

**Table 1:** Variant numbers from whole-exome data following filtering cascade

*Filter*	*Variants*	*Genes*
Start	357 699	17 704
I: Confidence	114 411	15 605
II: Common variants	20 104	5773
III: Predicted deleterious	880	558
IV: Genetic analysis	129	58
V: Biological context (peripheral neuropathy)	1	1
VI: Biological context (bronchiectasia^a^)	3	3

Filtering cascade as implemented in Ingenuity Variant Analysis, detailed descriptions of the filtering parameters are given in Supplementary Information.

a 10 variants in three genes were subsequently removed due to their location within segmental duplications.
