# Supplementary material for: The use of whole-exome sequencing to disentangle complex phenotypes
Source: Eur J Hum Genet. 2015 Jun 10;24(2):298–301. doi: 10.1038/ejhg.2015.121 (PMC4717198; doi:10.1038/ejhg.2015.121)

Supplementary table 1: Results of multipoint linkage analysis, regions with LOD>2.0


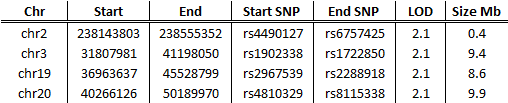


Supplemental table 2: Sequencing quality metrics


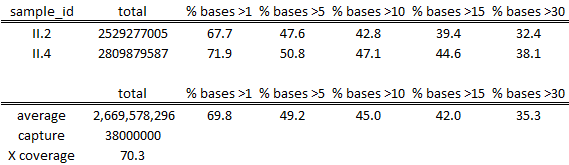


Supplementary figure 1: Sanger sequencing electrophoretograms for *IL33* intronic variant rs10975521; transcript NM_033439.3: c612+15T>A at position chr9.hg19:g.6254568 in pedigree
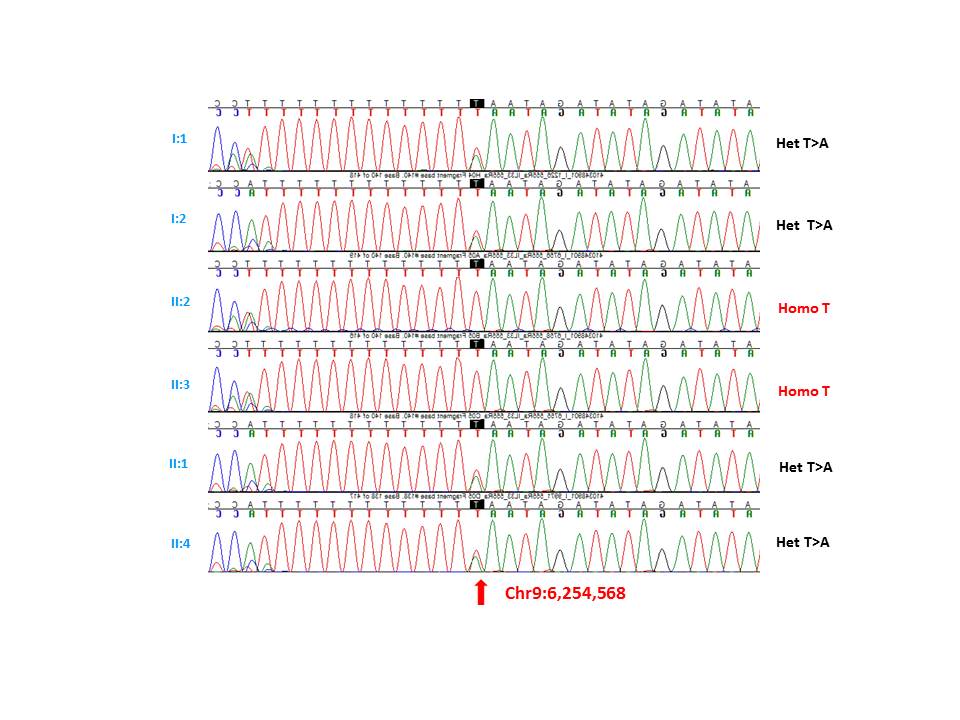


Supplementary figure 2: Multipoint parametric linkage plot for pedigree.


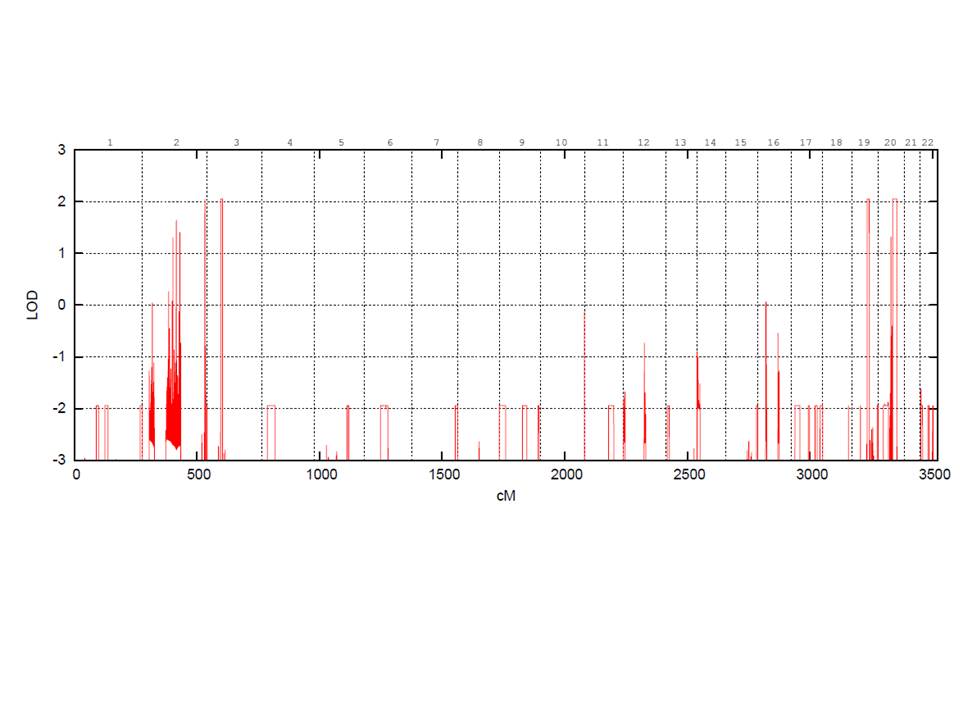

Supplement: Supplementary Information [file ejhg2015121x1.doc]
